# Effects of low light on photosynthetic properties, antioxidant enzyme activity, and anthocyanin accumulation in purple pak-choi (*Brassica campestris* ssp. *Chinensis* Makino)

**DOI:** 10.1371/journal.pone.0179305

**Published:** 2017-06-13

**Authors:** Hongfang Zhu, Xiaofeng Li, Wen Zhai, Yang Liu, Qianqian Gao, Jinping Liu, Li Ren, Huoying Chen, Yuying Zhu

**Affiliations:** 1 School of Agriculture and Biology, Shanghai Jiaotong University, Shanghai, China; 2 Shanghai Key Lab of Protected Horticultural Technology, Horticultural Research Institute, Shanghai Academy of Agricultural Sciences, Shanghai, China; 3 College of Horticulture, Nanjing Agricultural University, Nanjing, China; Northwest Agriculture and Forestry University, CHINA

## Abstract

Anthocyanins are secondary metabolites that contribute to red, blue, and purple colors in plants and are affected by light, but the effects of low light on the physiological responses of purple pak-choi plant leaves are still unclear. In this study, purple pak-choi seedlings were exposed to low light by shading with white gauze and black shading in a phytotron. The responses in terms of photosynthetic properties, carbohydrate metabolism, antioxidant enzyme activity, anthocyanin biosynthetic enzyme activity, and the relative chlorophyll and anthocyanin content of leaves were measured. The results showed that chlorophyll b, intracellular CO_2_ content, stomatal conductance and antioxidant activities of guaiacol peroxidase, catalase and superoxide dismutase transiently increased in the shade treatments at 5 d. The malondialdehyde content also increased under low light stress, which damages plant cells. With the extension of shading time (at 15 d), the relative chlorophyll a, anthocyanin and soluble protein contents, net photosynthetic rate, transpiration rate, stomata conductance, antioxidant enzyme activities, and activities of four anthocyanin biosynthetic enzymes decreased significantly. Thus, at the early stage of low light treatment, the chlorophyll b content increased to improve photosynthesis. When the low light treatment was extended, antioxidant enzyme activity and the activity of anthocyanin biosynthesis enzymes were inhibited, causing the purple pak-choi seedlings to fade from purple to green. This study provides valuable information for further deciphering genetic mechanisms and improving agronomic traits in purple pak-choi under optimal light requirements.

## Introduction

Light is one of the most important environmental factors and plays a critical function in plant development and metabolism [1,2]. Additionally, light is indispensable for photosynthesis and photomorphogenesis. Low light is a pervasive abiotic stress in plant breeding and cultivation due to light block from horticulture facilities, clouds and snow. Low light was shown to substantially affect the agronomic traits of plants and inhibit physiological metabolic processes, including photosynthesis and antioxidant characteristics, as well as carbon and nitrogen fixation [3–6]. It causes slow growth, decrease of leaf weight and flower bud number. Furthermore, this stressor reduces sugar and starch contents in eggplant, grape and rice [7–9], and changes the coloration and extends the maturity time in cherry [10].

Chlorophyll is an important pigment involved in absorbing, transmitting and converting solar energy into electrochemical energy [11]. It was reported [12] that low light-tolerant hybrid rice -exhibited a higher content of chlorophyll b following exposure to low light. Low light negatively affects stomata conductance and results in enhanced concentration of intercellular CO_2_ in rice leaves [13,14]. Moreover, stomata conductance and photosynthetic efficiency under low light decreases by the number of 24.31% and 79.84%, respectively compared to that of natural light [15].

Antioxidant metabolism plays an important role in protecting plants from a wide variety of environmental stresses, such as drought, extreme temperatures, pollutants, ultraviolet radiation and high levels of light [16,17]. Enhancement of antioxidant defense in plants can thus increase tolerance to different stresses. Antioxidants include the enzymes peroxidase (POD), catalase (CAT), ascorbate peroxidase (APX) and superoxide dismutase (SOD) [18]. Analyses of membrane lipid peroxidation in peach fruit showed that decreasing the light intensity decreased CAT, G-POD and APX activity but increased malondialdehyde (MDA) content with more cell membrane damage [6,19].

Pak-choi (*Brassica campestris* ssp. *Chinensis* Makino L.) originated from China is one of the most important vegetables worldwide in terms of its planting areas and annual yields. Purple pak-choi contains high levels of light-dependent anthocyanin in its leaves. This plant is very popular in China, Japan and surrounding countries. Anthocyanins, a class of secondary metabolites, contribute to the red, blue, and purple colors in flowers, fruits, and leaves [20]. They also act as antioxidants and protect DNA and the photosynthetic apparatus from damage due to high radiation fluxes [21]. Additionally anthocyanin protects plants against cold and drought stress [22]. Anthocyanin is accumulated in response to light in the seedlings of mustard and tomato. Phytochrome is an important photoreceptor controlling the accumulation of anthocyanins [23–26]. However, how phytochrome regulates anthocyanin and other key enzymes under low light remains unclear.

**Fig 1:**
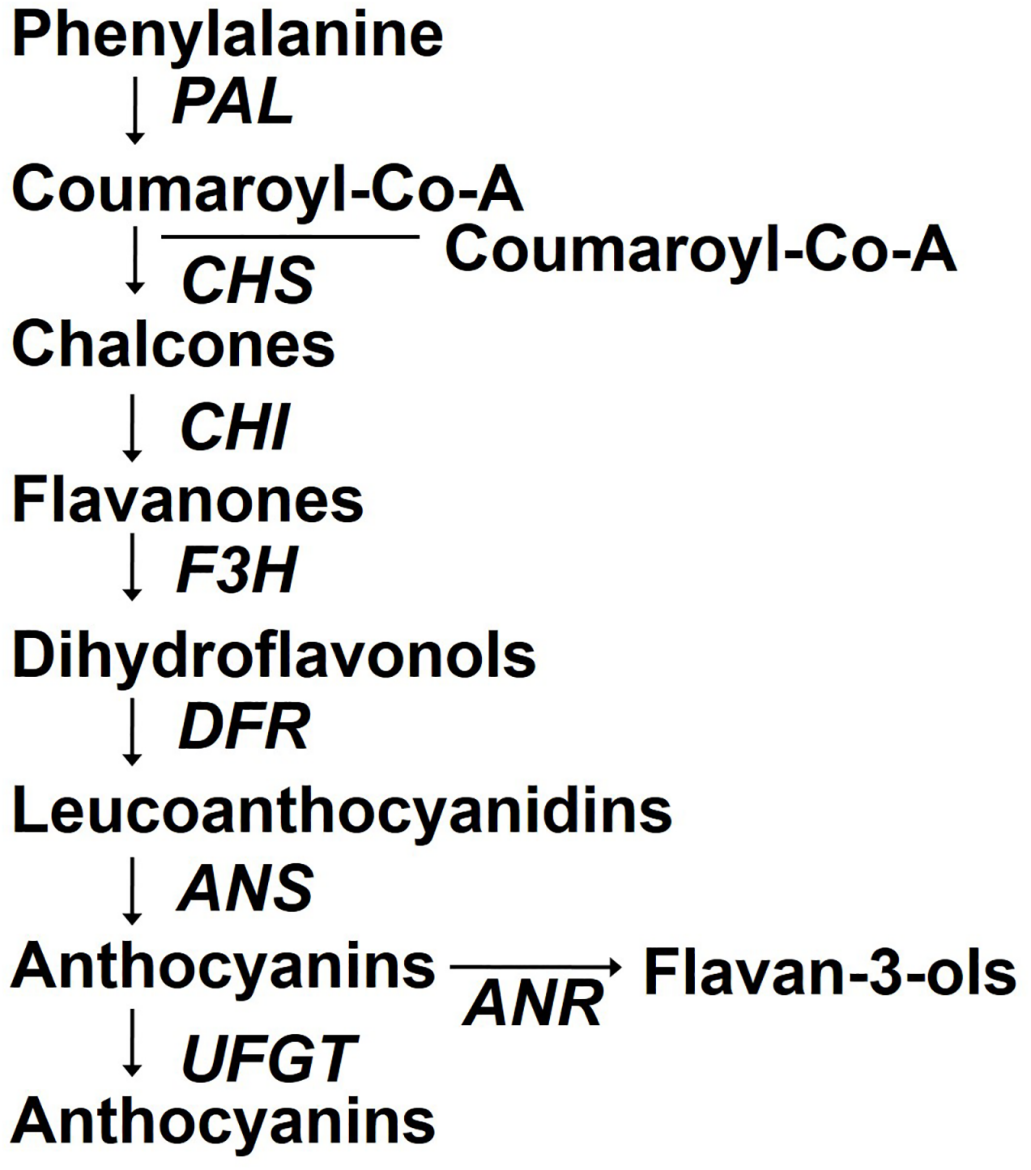
Diagram of the flavonoid pathway. The enzymes for each step are italicized, with the following enzymes required for anthocyanin biosynthesis: chalcone synthase (*CHS*), chalcone isomerase (*CHI*), flavanone-3-hydroxylase (*F3H*), dihydroflavonol-4-reductase (*DFR*), anthocyanidin synthase (*ANS*), anthocyanidin reductase (*ANR*) and UDP-glucose: flavonoid-3-O-glycosyltranferase (*UFGT*).

Anthocyanin is synthesized via a branch of the phenylpropanoid pathway, i.e., the flavonoid pathway (Fig 1) [27]. Anthocyanin biosynthesis consists of sequential reactions leading to the production of different anthocyanins. The gene structure of 73 anthocyanin biosynthetic genes was identified in *B*. *rapa* [28]. These gene expression analyses showed that almost all late biosynthetic genes of anthocyanin were highly up-regulated in all purple leaves of *Brassica* [29]. The key enzymes in the anthocyanin biosynthetic pathway include chalcone synthase (*CHS*), chalconeisomerase (*CHI*), flavanone 3-hydroxylase (*F3H*), dihydroflavonol-4-reductase (*DFR*), leucoanthocyanidin oxygenase (*LDOX*), anthocyanidin synthase (*ANS*), and anthocyanidin reductase (*ANR*). *CHI* is the first identified key enzyme in the flavonoid metabolic pathway [30], while *CHS* is the first enzyme in the pathway [31].

Cominelli et al. [32] investigated different light treatments and found that the activities of *ANS* and *ANR* in *Arabidopsis* were related to their gene expression level. The regulation of anthocyanin accumulation under different light levels was shown to be due to transcriptional control or transcription factors [33–37].

The lowest light levels caused death and decreased anthocyanin content in *Anacampseros rufescens*, maize and perilla [38–40]. The shading of stems and leaves of *Eustoma grandiflorum* resulted in a significant anthocyanin reduction in petal color [41], while incubation in complete darkness was beneficial to the nutritional quality of the brassica sprouts [42]. When *B*. *rapa* was exposed to low light, the levels of phenolics and shoot biomass were reduced [43]. Still, a comprehensive study of physiological change after low light treatment is lacking. Thus, we investigated the responses of various plant parameters, such as photosynthesis, chlorophyll, and the activities of anthocyanin biosynthetic and antioxidant enzymes, in purple pak-choi under low light stress by shading the plants in a phytotron. Moreover, the examination of anthocyanin accumulation in this purple plant under different low light intensities provides invaluable guidance for artificially supplementing light intensity in agricultural facilities.

## Materials and methods

### Ethics statement

This study was carried out in a phytotron. No specific permissions were required. The study did not involve any endangered or protected species.

### Plant material and treatments

The variety of purple pak-choi (*Brassica campestris* ssp. *Chinensis* Makino L. "ziyi") was selected by the Horticultural Research Institute of Shanghai Academy of Agricultural Sciences, China. Initially 192 purple pak-choi seeds were sown in 12 plastic plates growing at a temperature of 28/15°C day/night in a greenhouse on October 20, 2015. Plants were watered and fertilized daily with a half-strength Hoagland nutrient solution. The low light treatments were started when the plant had three expanded leaves (four weeks after sowing on November 17). They were transferred and maintained in a phytotron with the temperature of 28/15°C day/night and 60% humidity. They were divided into four groups and were exposed to low light treatment as follows (Table 1): (1) normal light (NL, 1000 μmol m^–2^ s^–1^), (2) low light 1 (TL1, 750 μmol m^–2^ s^–1^), (3) low light 2 (TL2, 500 μmol m^–2^ s^–1^), and (4) low light 3 (TL3, 250 μmol m^–2^ s^–1^). The light intensity was measured by a Specbos 4001 (JETI Company, Germany). The experiment was carried out in triplicate, and approximately 100 plants were used in each replicate.

**Table 1:** Low light treatment of purple pak-choi seedlings in a phytotron.

Category	Rate of light transmittance (%)	Treatment	Illumination intensity (μmol m^–2^ s^–1^)
**NL**	100	Normal illumination	1000
**TL1**	75	A layer of white gauze	750
**TL2**	50	A layer of black shading with 50% transmittance	500
**TL3**	25	A layer of black shading with 25% transmittance	250

### Relative pigment levels

Twenty-milligram samples of purple pak-choi leaves were incubated with 10 ml of 95% ethanol in the dark for 24 h and mixed by vortexing for 30 s after 12 h. The relative chlorophyll and carotenoid levels were measured with a spectrophotometer (DU 730, Beckman Coulter, Inc., Brea, CA, USA) at 649, 665, and 470 nm, and then the amount of chlorophyll a, chlorophyll b, and carotenoid was calculated using formulas 2.1, 2.2 and 2.3 [44]:

$$Chlorophyll\;a=13.95\;A_{665}-6.88A_{649}$$ (2.1)

$$Chlorophyll\;b=24.96A_{649}-7.32A_{665}$$ (2.2)

$$Carotenoid=(1000A_{470}-2.05\times chlorophyll\;a-114.8\times chlorophyll\;b)/245$$ (2.3)

The total anthocyanin content (TAC) of purple pak-choi was quantified with a modified pH differential method (AOAC official method 2005.2) [45,46]. The TAC was derived using cyanidin-3-glucoside, which has a molar extinction coefficient of 26,900 L cm^-1^ mol^-1^ and a molecular weight of 449.2 g mol^-1^. The results are expressed as milligrams of cyanidin-3-glucoside equivalent per gram of fresh weight sample. Twenty-milligram leaf samples were incubated with 10 ml buffer (95% ethanol and 1.5 mol l^-1^ HCl (v/v) 85:15) at room temperature in the dark for 24 h. Then, 1 ml of leaf supernatant was mixed separately with 2 ml of 0.025 M KCl buffer at pH 1.0 and 0.4 M sodium acetate (NaAc) buffer at pH 4.5. Absorbance was read by a nucleic acid/protein analyzer (Beckman Coulter, Inc., USA) at 536 nm and at 700 nm in the pH 1.0 and pH 4.5 buffers, respectively. TAC was calculated with the following equation (2.4):

$$A=\left(A_{536}-A_{700}\right)\;\mathrm{pH}1.0-(A_{536}-A_{700})\;\mathrm{pH}4.5$$ (2.4)

### Leaf gas exchange

Leaf gas exchange was measured on a fully developed leaf from the middle of each seedling at 9:30 AM after 5 d, 10 d and 15 d of low light treatment by a Li-6400 Portable Photosynthesis System (Li-Cor Inc., Lincoln, NE, USA). The CO_2_ assimilation rate or net photosynthetic rate (*P*_*n*_), stomatal conductance (*G*_*s*_), intercellular carbon dioxide (*C*_*i*_) and transpiration rate (*T*_*r*_) of purple pak-choi leaves were analyzed. After measurement, the largest leaves from each group in the same position were harvested. Three biological replicates were frozen immediately in liquid nitrogen and stored at -80°C for further analysis.

### Quantification of MDA and soluble protein

The frozen leaf samples were ground to determine the MDA and soluble protein content. As described by Jiang and Zhang [47], the amount of MDA, which represents lipid peroxidation was calculated by its molar extinction coefficient (155 mM^−1^ cm^−1^) in the thiobarbituric acid reaction. Total soluble protein content was measured using the Bradford reagent [48].

### Antioxidant enzyme activity assay

For the enzyme assays, 0.2 g of leaf samples were ground in 3 ml of ice-cold 25 mM HEPES buffer (0.2 mM EDTA, 2 mM ASA, and 2% PVP, pH 7.8). The homogenates were centrifuged at 4°C for 20 min at 12,000 g, and the supernatants were used to determine the enzymatic activities. The G-POD activity was measured by the modified method of Cakmak [49]. The reaction mixture had 25 mM phosphate buffer (pH 7.0), 0.05% guaiacol, 1.0 mM H_2_O_2_ and 100 μl of enzyme extract. The increase in absorbance at 470 nm caused by guaiacol oxidation (E = 26.6 mM cm^-1^) was used to determine the G-POD activity. CAT was assayed as described by Durner and Klessing [50], and the activity was determined as a decrease in the absorbance at 240 nm for 1 min following the decomposition of H_2_O_2_. APX was measured by monitoring the rate of ascorbate oxidation at 290 nm as described by Nakano and Asada [51]. SOD activity was measured in a mixture of 50 mM phosphate buffer (pH 7.8), 0.1 mM EDTA, 13 mM methionine, 75 μM nitroblue tetrazolium (NBT), 2 μM riboflavin, and 50 μl of enzyme [52]. One unit of SOD activity was defined as the amount of enzyme required to inhibit 50% of the p-nitro blue tetrazolium chloride reduction at 560 nm.

### Anthocyanin biosynthetic enzyme activity assay

For these assays, 0.2 g of leaf samples were ground in 2 ml of ice-cold 25 mM HEPES buffer (pH 7.4) containing 0.2 mM EDTA, 2 mM AsA, and 2% PVP. The homogenates were centrifuged at 4°C for 20 min at 12,000 g, and the supernatants were used to determine the enzymatic activities. The activity of 6 anthocyanin biosynthetic enzymes, *CHS*, *CHI*, *F3H*, *DFR*, *ANS* and *ANR*, were assayed using an ELISA Kit (U.S.A TSZ Biological Trade Co., Ltd.) according to the manufacturer’s instructions. This experimental method was based on a laboratory protocol deposited in protocols io, which was obtained from doi:dx.doi.org/10.17504/protocols.io.h2mb8c6.

### Statistical analysis

Statistical Product and Service Solutions (SPSS, version 20, IBM Corporation, U.S.A) was used to performed analysis of variance (ANOVA). The physiological variables are presented as the mean ± standard deviation (SD), with a minimum of three replicates. Differences between the control and treatments were considered significant at *p* = 0.05. Significance between treatments was determined by Duncan’s *t*-test. The data were plotted using Origin 7.5 software (Origin Lab, Northampton, MA, USA).

## Results

### Analysis of relative pigment levels

**Fig 2:**
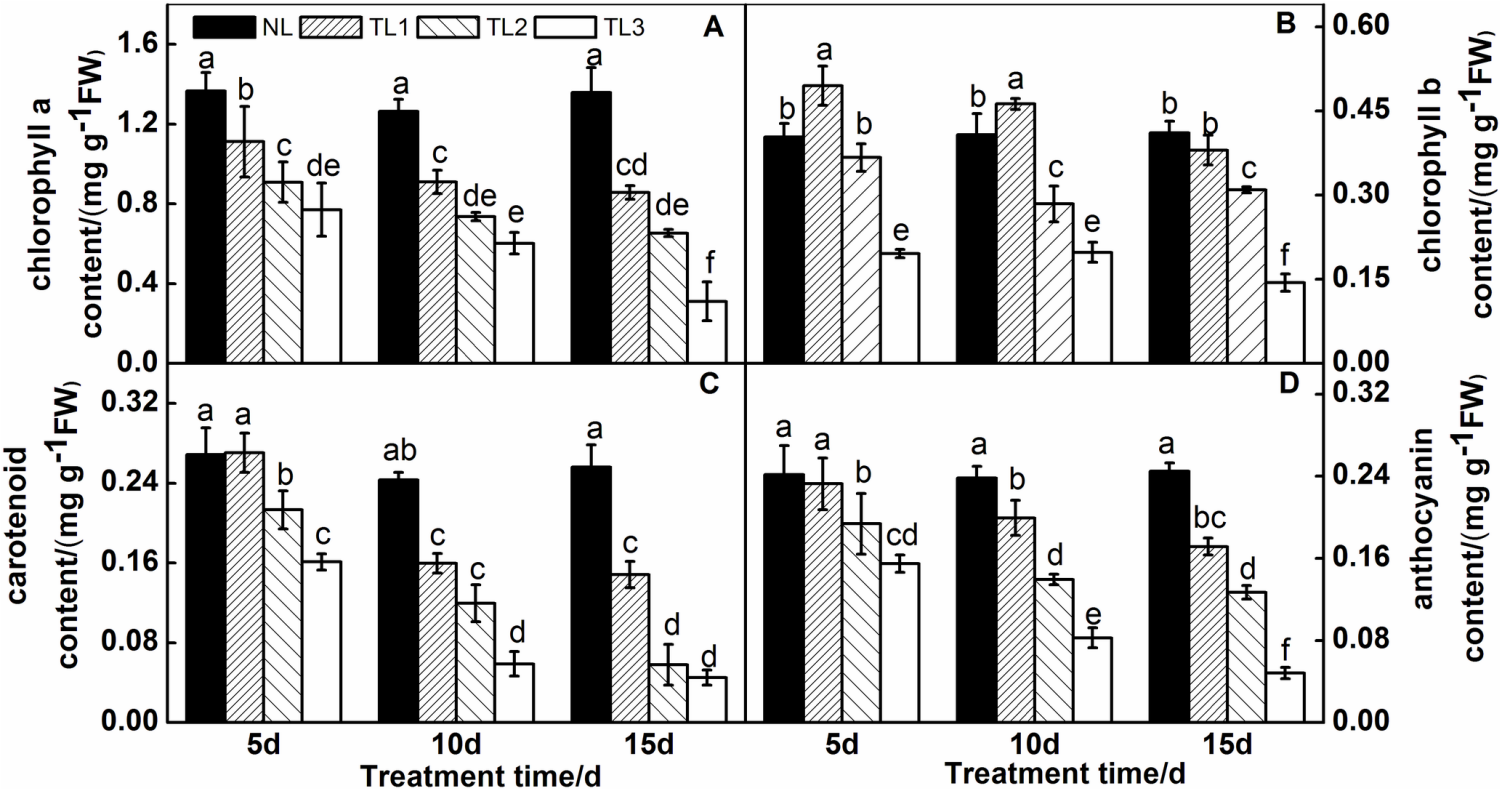
Effects of low light treatment on chlorophyll a (A), chlorophyll b (B), carotenoid (C) and anthocyanin (D) content in leaves of purple pak-choi seedlings. The data are the mean of three replicates, and SDs are shown as *vertical bars*. The means marked with different letters indicate significant differences between treatments at *p*<0.05, as determined with Duncan’s multiple range test. Different lower-case letters at each time point indicate significant differences between treatments. FW is the abbreviation of fresh weight.

Low light stress had significant effects on the anthocyanin, carotenoid and relative chlorophyll contents of purple pak-choi (Fig 2). The chlorophyll a contents were significantly reduced after exposure to low light stress for 5 d, 10 d and 15 d (Fig 2A). The chlorophyll a content of TL1, TL2 and TL3 was significantly lower than that in the group exposed to NL by 18.59%, 33.45% and 51.19%, respectively, at 5 d; significantly decreased by 27.95%, 41.71% and 52.35%, respectively, at 10 d; and significantly declined by 40.36% 51.91% and 77.10%, respectively, at 15 d.

The chlorophyll b content in TL1 leaves was 22.53% and 13.39% higher than that in NL group at 5 d and 10 d, respectively (Fig 2B). However, when exposed to low light for 15 d, the chlorophyll b content of TL1 clearly decreased by 7.91%. The chlorophyll b content of TL2 and TL3 leaves greatly decreased at 10 d and 15 d by 9.11% and 51.51% respectively, compared with that of NL leaves at 5 d; the content was 30.30% and 51.42% lower, respectively, at 10 d; and 24.66% and 64.95% lower, respectively, at 15 d.

The carotenoid content in TL1 did not differ significantly from that in the NL group at 5 d (Fig 2C), but TL2 and TL3 were 20.03% and 40.08% lower than NL, respectively. The carotenoid content in TL1, TL2 and TL3 decreased by 34.31%, 50.81% and 75.82%, respectively, at 10 d; at 15 d, it decreased by 46.68%, 77.35% and 82.42%, respectively.

The anthocyanin content decreased from 5 d to 15 d under low light stress (Fig 2D). The anthocyanin content in TL1, TL2 and TL3 was 3.69%, 19.81% and 35.93% lower than that in NL at 5 d, respectively; 16.24%, 41.51% and 65.34% lower than that in NL at 10 d, respectively; and 29.91%, 48.17% and 80.31% lower than that in NL at 15 d, respectively.

### Leaf gas exchange analysis

**Fig 3:**
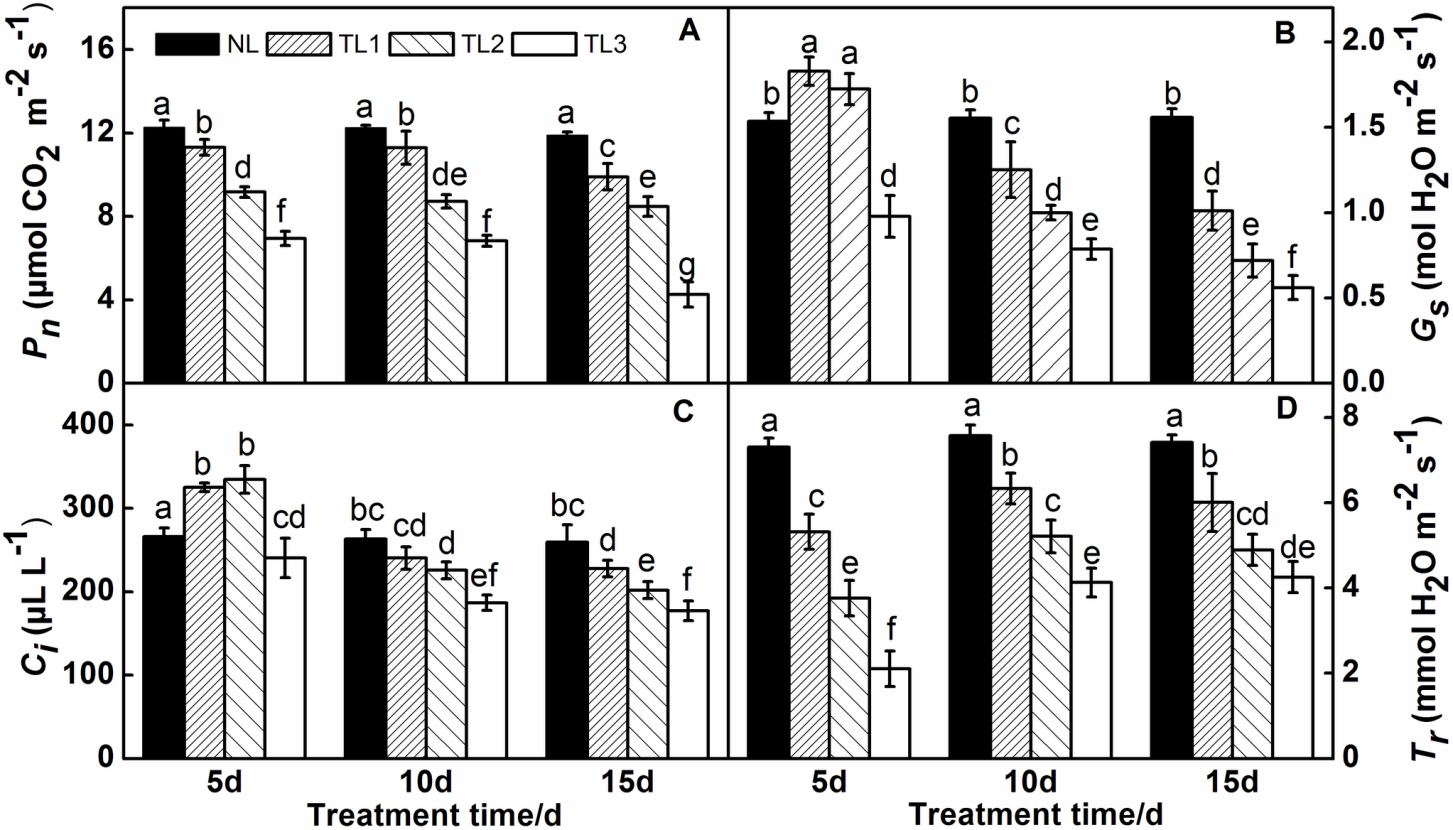
Effects of low light treatment on photosynthetic rate *(P*_*n*_) (A), stomatal conductance (*G*_*s*_) (B), intercellular CO_2_ concentration (*C*_*i*_) (C), and transpiration rate (*T*_*r*_) (D) of purple pak-choi seedlings. The data are the mean of three replicates, with SDs shown as *vertical bars*. The means marked with different letters indicate significant differences between treatments at *p*<0.05, as determined with Duncan’s multiple range test. Different lower-case letters at each time point indicate significant differences between treatments.

*P*_*n*_, *G*_*s*_, *C*_*i*_ and *T*_*r*_ were significantly influenced by low light treatment (Fig 3). The rate of CO_2_ assimilation (*P*_*n*_) decreased sharply with low light treatment (Fig 3A). Five days after low light treatment, the *P*_*n*_ of TL1, TL2 and TL3 dramatically decreased by 7.90%, 25.33% and 43.47% relative to that of NL, respectively; at 10 d, the *P*_*n*_ decreased by 7.91%, 28.89% and 44.34% relative to that of NL, respectively; and the *P*_*n*_ was 16.84%, 28.79% and 64.23% lower than that in NL at 15 d, respectively. However, there were no significant differences between different treatment times for the same treatment.

*G*_*s*_ in TL1 and TL2 was increased (Fig 3B) and the leaf stomata opened prominently when leaves were exposed to low light for 5 d, *G*_*s*_ was increased by 19.11% (TL1) and 18.83% (TL2) relative to that in NL. However, *G*_*s*_ in TL3 was 36.35% lower than that in NL. *G*_*s*_ in TL1, TL2 and TL3 was decreased by 12.94%, 35.60% and 49.38% at 10 d, respectively, and by 35.16%, 53.90% and 64.03% at 15 d, respectively. The changes in *C*_*i*_ were similar to those in *G*_*s*_ as the *C*_*i*_ value of TL1 and TL2 increased transiently after 5 d (Fig 3C); then, at 10 d and 15 d, the *C*_*i*_ values of all treatments were significantly decreased.

In terms of transpiration, the *T*_*r*_ of TL1, TL2 and TL3 showed a prominent decrease at all treatment times (5 d, 10 d, and 15 d) under low light stress (Fig 3D). The *T*_*r*_ was sharply lower than in NL, with 27.22%, 48.56% and 71.16% in TL1, TL2 and TL3 lower than NL at 5 d, respectively; 16.41%, 31.18% and 45.44% lower than NL at 10 d, respectively; and 19.00%, 34.00% and 42.65% lower than NL at 15 d, respectively.

### MDA and soluble protein analysis

**Fig 4:**
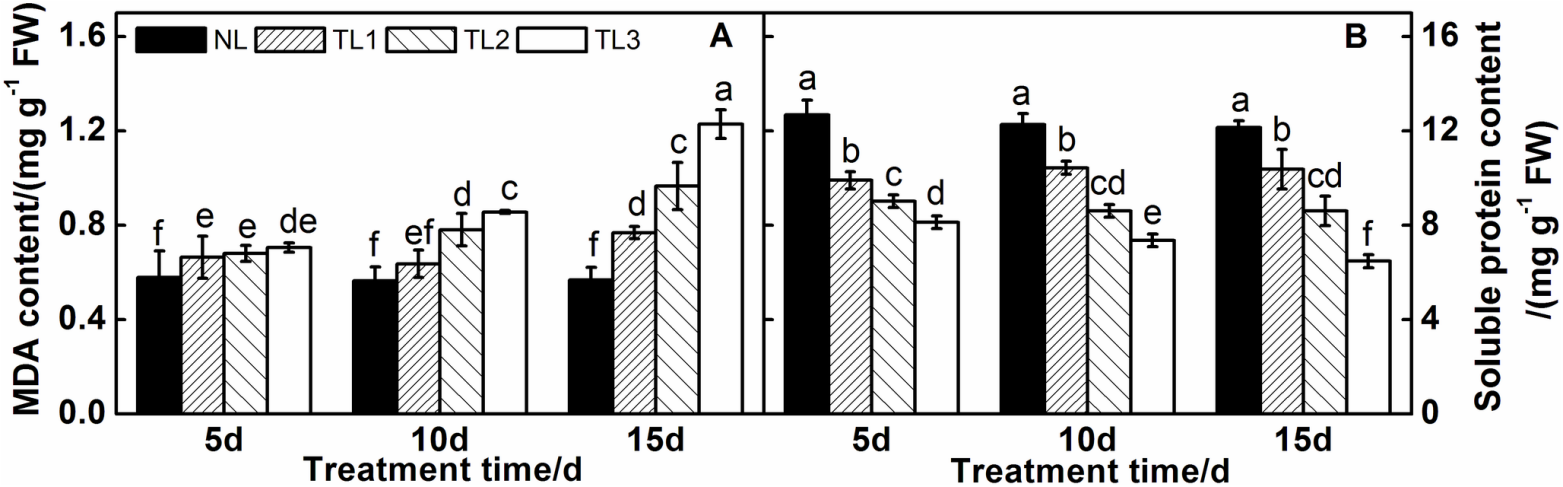
Effects of low light treatment on the MDA (A) content and the soluble protein (B) content in leaves of purple pak-choi seedlings. The data are the mean of three replicates, with SDs shown as *vertical bars*. The means marked with different letters indicate significant differences between treatments at *p*<0.05, as determined with Duncan’s multiple range test. Different lower-case letters at each time point indicate significant differences between treatments. FW is the abbreviation of fresh weight.

When purple pak-choi plants were exposed to low light stress, the MDA content increased in this experiment (Fig 4A). Under low light stress for 5 d, the MDA contents of TL1, TL2 and TL3 were higher than those in NL, but these differences were not statistically different. At 10 d and 15 d, the MDA content in these groups increased significantly, and the same treatment was significantly different at different treatment times. The MDA content of TL1, TL2 and TL3 increased significantly by 0.77 mg g^-1^ FW, 0.97 mg g^-1^ FW and 1.23 mg g^-1^ FW, respectively at 15 d. However, the soluble protein content decreased with time (Fig 4B). Compared to that of NL, the soluble protein content of TL1, TL2 and TL3 was 21.90%, 28.86% and 35.88% lower at 5 d, respectively; 14.93%, 29.87% and 39.98% lower at 10 d, respectively; and 14.59%, 29.18% and 46.69% lower at 15 d, respectively. There were significant differences between different treatments at 10 and 15 d, but the same treatment did not differ significantly at different treatment times.

### Antioxidant enzyme analysis

**Fig 5:**
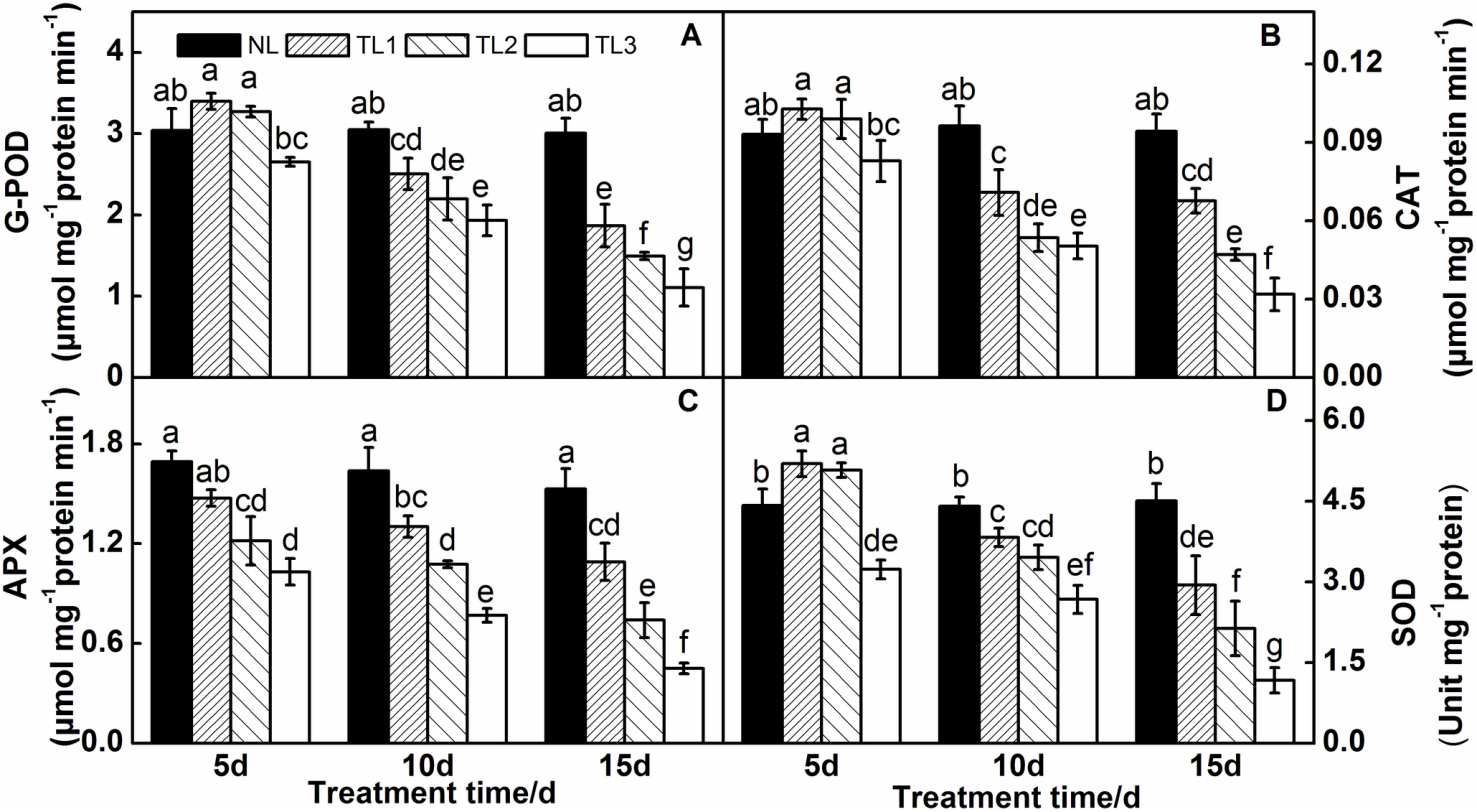
Effects of low light on guaiacol peroxidase (G-POD) (A), catalase (CAT) (B), ascorbate peroxidase (APX) (C) and superoxide dismutase (SOD) (D) activities in the leaves of purple pak-choi seedlings. The data are the mean of three replicates, with SDs shown as *vertical bars*. The means marked with different letters indicate significant differences between treatments at *p*<0.05, as determined with Duncan’s multiple range test. Different lower-case letters at each time point indicate significant differences between treatments.

Low light stress resulted in significant changes of enzymatic activities of G-POD, CAT, APX, and SOD (Fig 5). For both the TL1 and TL2 leaves, the enzymatic activities of G-POD, CAT and SOD increased clearly after 5 d of the low light treatment (Fig 5A, 5B and 5D); however, the activities of these three enzymes decreased markedly after 10 d. G-POD activity decreased by 34.75% (TL1) and 50.31% (TL2), the CAT enzyme activity decreased by 28.26% (TL1) and 50.15% (TL2), and the SOD enzyme activity decreased by 34.75% (TL1) and 52.57% (TL2) relative to those of the NL leaves after 15 d. In contrast to TL1 and TL2, the G-POD, CAT and SOD enzyme activities decreased gradually from 5 d to 15 d in TL3.

For all the treatments, the changes in APX enzyme activities showed the same patterns as they were lower in TL1, TL2 and TL3 than those in NL leaves at 5, 10 and 15 d (Fig 5C). The APX activities of TL1, TL2 and TL3 leaves, decreased markedly by 14.82%, 28.09% and 39.11% at 5 d, respectively; by 20.36%, 34.29% and 53.03% at 10 d, respectively; and by 28.59%, 51.57% and 70.56% at 15 d, respectively.

### Anthocyanin biosynthetic enzyme analysis

**Fig 6:**
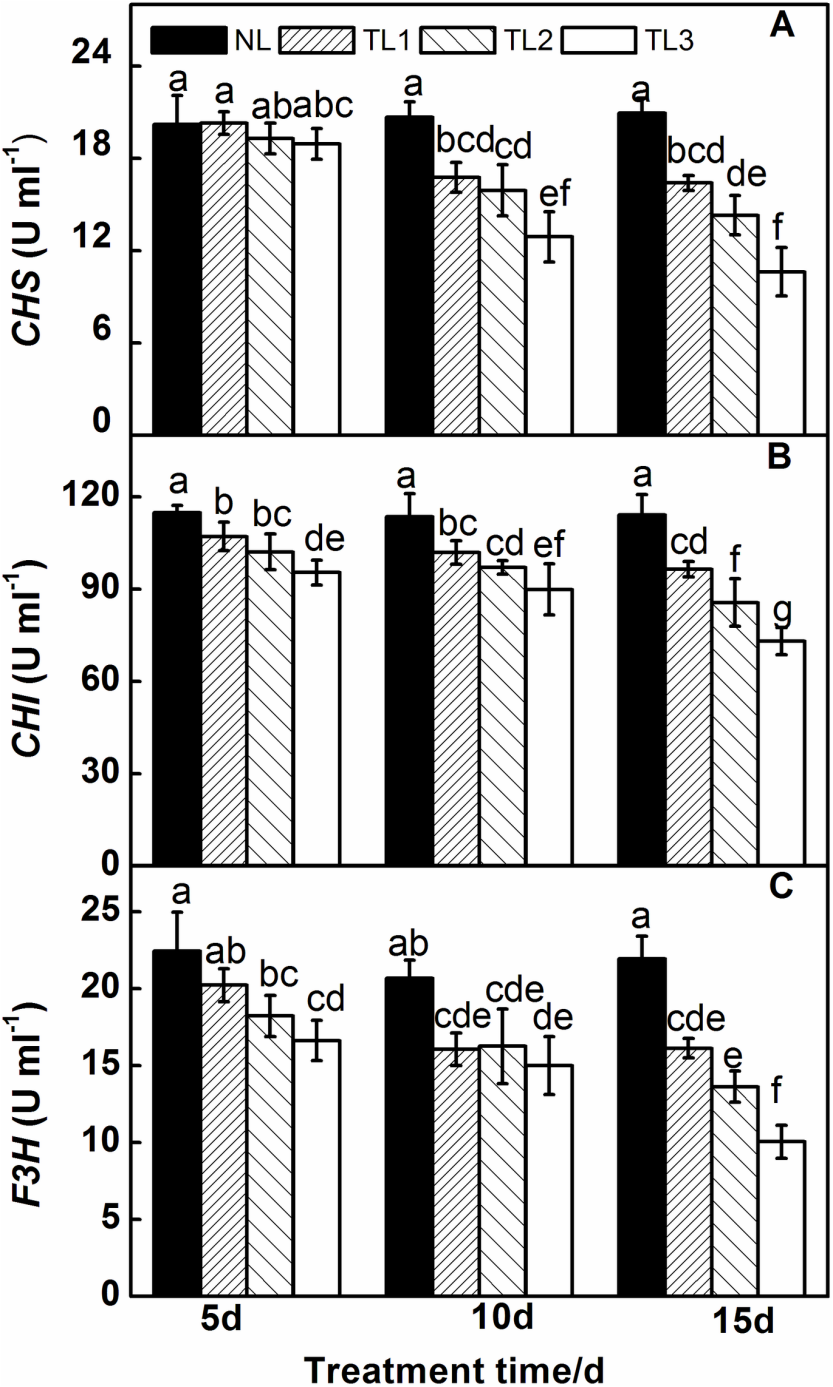
Effects of low light on chalcone synthase (*CHS*), chalcone-flavanone isomerase (*CHI*), flavanone-3- hydroxylase hydroxylation (*F3H*), activities in the leaves of purple pak-choi seedlings. The data are the means of three replicates, with SDs shown as *vertical bars*. The means marked with different letters indicate significant difference between treatments at *p*<0.05, as determined with Duncan’s multiple range test. Different lower-case letters at each time point indicate significant differences between treatments.

Three key enzymes (*CHS*, *CHI*, *F3H*) in the anthocyanin biosynthetic pathway, affected significantly under low light stress, are shown in Fig 6. The *CHS* activity in NL, TL1, TL2 and TL3 leaves did not differ significantly at 5 d (Fig 6A), while it decreased slowly at 10 d. The activity was 21.70% (TL1), 31.72% (TL2) and 49.28% (TL3) lower than that in NL at 15 d. The *CHI* activity in TL1, TL2 and TL3 leaves was significantly lower than that in NL leaves at 5 d, 10 d and 15 d (Fig 6B). The TL2 and TL3 treatments were 11.75% and 18.69% lower at 15 d, respectively, than that at 10 d.

The *F3H* activity in NL, TL1, TL2 and TL3 leaves was 22.44 U ml^-1^, 20.22 U ml^-1^, 18.22 U ml^-1^ and 16.62 U ml^-1^ at 5 d under low light stress, respectively (Fig 6C). At 10 d, the *F3H* activity of TL1, TL2 and TL3 leaves decreased by 22.28%, 21.32% and 37.37% relative to that of NL leaves, although *F3H* activity was not significantly different among TL1, TL2 and TL3. However, the *F3H* activity in NL, TL1, TL2 and TL3 leaves at 15 d was 21.93 U ml^-1^, 16.13 U ml^-1^, 13.26 U ml^-1^ and 10.04 U ml^-1^, respectively. The *F3H* activity in TL1, TL2 and TL3 was significantly lower than that in NL. In our experiment, the trend of *DFR* activity in all treatments was similar to that observed for *F3H* activity, and the *ANS* activity of treated leaves was significantly lower than that of NL leaves exposed to low light stress at 15 d. However, the *ANR* activity of NL, TL1, TL2 and TL3 leaves did not differ significantly at 5 d, 10 d or 15 d.

## Discussion

Photosynthetic pigments play an important role in photosynthesis as they can assimilate and transfer light energy. Therefore, the pigments contents directly affect the photosynthetic efficiency. Chlorophylls are one of the most important pigments and represent a significant index of photosynthetic capacity [53]. In general, chlorophyll content will decrease after exposure to low light stress. In our study, chlorophyll a content was decreased when purple pak-choi was exposed to low light (TL1, TL2 and TL3). In addition, as the duration of exposure increased, the chlorophyll a content exhibited increasingly serious damage. However, the level of chlorophyll b, which absorbs diffuse light with a short wavelength, increased temporarily at 5 d and 10 d, but decreased at 15 d in response to low light stress. Chlorophyll b is responsible for transferring light energy in photosynthesis and could capture more energy to improve the utilization efficiency under low light stress.

Our results indicated that the chlorophyll a content under low light stress was decreased in purple pak-choi resulting in photosynthetic damage, but the chlorophyll b content was increased to resist low light stress. Ma et al. [54] found that the chlorophyll a content did not change under low light, while the chlorophyll b content increased. Lakshmi and Singh [55,56] stated that chlorophyll a and chlorophyll b both increased under low light stress, whereas Bell [38] found that the chlorophyll content of *Agrostis stolonifera* decreased after long-term exposure to low light stress, and so that the plants exhibited etiolating, withering and dying when the light shade was to 95%.

Our results were consistent with a previous study that found the carotenoid and anthocyanin contents decreased under low light stress [57]. Anthocyanin is a specific characteristic of purple pak-choi. The reduction of anthocyanin content led to green plants or severe etiolation of plants. In our experiment, the carotenoid and anthocyanin levels were reduced to 0.048 mg g^-1^ FW and 0.045 mg g^-1^ FW_,_ respectively, when the shade was more than 75% (TL3) at 15 d, and the color of the purple pak-choi became light green. Nielsen [58] reported that high light intensity promoted anthocyanin synthesis and accumulation so that the purple basil leaves were darker purple than those grown under low light. Shading pears and apples during cold conditions for 2 d reduced the accumulation of anthocyanin and increased their photosensitivity [59].

Photosynthesis is the fundamental physiological process that provides energy and carbon assimilation for plant growth [60], but it is often inhibited and damaged due to its sensitivity to low light stress [61]. In the present study, the leaf photosynthetic parameters *P*_*n*_, *G*_*s*_, *C*_*i*_ and *T*_*r*_ showed significant responses to low light levels. The *P*_*n*_ and *T*_*r*_ of TL1, TL2 and TL3 leaves were significantly decreased after exposure to low light, whereas the *C*_*i*_ and *G*_*s*_ of TL1 and TL2 first showed an increase after 5 d and then decreased at 10 d and 15 d. The proportion of shading determined the extent of the decrease. Our results showed that the purple pak-choi seedlings receiving shade treatment exhibited impaired photosynthetic capacity due to reduced *P*_*n*_, *G*_*s*_, *C*_*i*_ and *T*_*r*_. In TL1 and TL2, *C*_*i*_ and *G*_*s*_ were increased to adapt to the low light environment in the short term and finally decreased to a level less than that in the NL plants. These results are consistent with the findings of Holmgren [62], who concluded that short-term shading caused elevated carbon dioxide levels in *Schefflera* seedling *diachyma* cells. Crookston et al [63] found that the *P*_*n*_ decreased by 38% under low light treatment in soybeans, which was the major cause of the decline in leaf photosynthetic rate. Fay and Knapp [64] also found that the *G*_*s*_ and *T*_*r*_ of soybean were decreased by half after exposure to shaded light for 9 min. However, Duli and Derrick [65] found that the decrease of *P*_*n*_ in cotton was not related to *G*_*s*_ and *T*_*r*_ under low light, instead the reduction of *P*_*n*_ resulted from the photosynthetic electron transfer capability. Therefore, the light responses in terms of photosynthesis were different among various species.

Proteins are required for biological activity, and thus the response to biotic or abiotic stress will undoubtedly be reflected in protein content and composition. We found that the soluble protein contents of TL1, TL2 and TL3 leaves were significantly lower than those in NL leaves at 5 d, 10 d and 15 d, and they decreased further as the shade increased, indicating that the accumulation of soluble protein was inhibited under low light stress leading to decreased production [56,66]. These findings were consistent with the results of Cockshull et al [67], who proposed that low light intensity was harmful to soluble proteins in tomato plants.

Low light stress causes different types and levels of damage to plant cells. One type involves the destruction of the membrane integrity for leaf blade cells, which leads to increased cell permeability and intracellular conductivity. MDA, which is produced during lipid peroxidation, is an important index of cell damage under stress. In the current study, the MDA content in purple pak-choi increased under low light stress, indicating that the degree of lipid peroxidation in the cell membrane is related to the duration of low light treatment and the degree of shaded light.

Oxidative stress is activated under biotic and abiotic stresses and results in the abundant production of reactive oxygen species (ROS) [68]. POD, CAT, APX and SOD can scavenge H_2_O_2_ [69]. Almeselmani et al [70] and Dai. et al. [71] reported that the amelioration of oxidation resistance occurs due to antioxidant enzyme activity. In the present study, the activities of G-POD, CAT, and SOD increased in TL1 and TL2 leaves after 5 d of low light stress, but decreased in TL1, TL2 and TL3 at 10 d and 15 d. In contrast, in all low light treatments, the APX activity decreased. Our results indicate that low light stress could produce ROS and increase the activity of antioxidant enzymes. However, antioxidant enzyme activity is inhibited when stress exceeds a certain degree, and plants suffer oxidative damage when the ROS are not eliminated. This finding is consistent with the results reported by Zhang and Marcelo [72,73].

Light requirements and low temperature stimulated a series of enzymes in the anthocyanin biosynthetic pathway [74,75]. Takos et al [27] reported that light was the key environmental factor leading to anthocyanin synthesis in red apples. Many reports have described the function of enzymes in anthocyanin biosynthesis and anthocyanin accumulation. For example, *DFR* played an important role in anthocyanin biosynthesis in strawberry fruit [76]. *CHS* and *DFR* were critical in the process of anthocyanin biosynthesis in mature red peach and nectarine fruit [77]. In bayberry fruit, *F3H*, *DFR* and *ANS* levels were highly correlated with anthocyanin biosynthesis [78]. The results of the present study suggested that the enzyme activities of *CHI*, *CHS*, *F3H*, which are involved in the anthocyanin biosynthetic pathway, were decreased under low light stress for 10 d and 15 d, leading to a decline of anthocyanin. However, *ANR* activity did not show significant changes in any of the low light treatments. Therefore, *ANR* may not be regulated by low light stress. Our studies of low light in purple pak-choi are consistent with results in *Arabidopsis thaliana* [72], *Perillafrutescens* [75], *Vitisvinifera* [79] and *Gerbera hybrida* [80].

**Fig 7:**
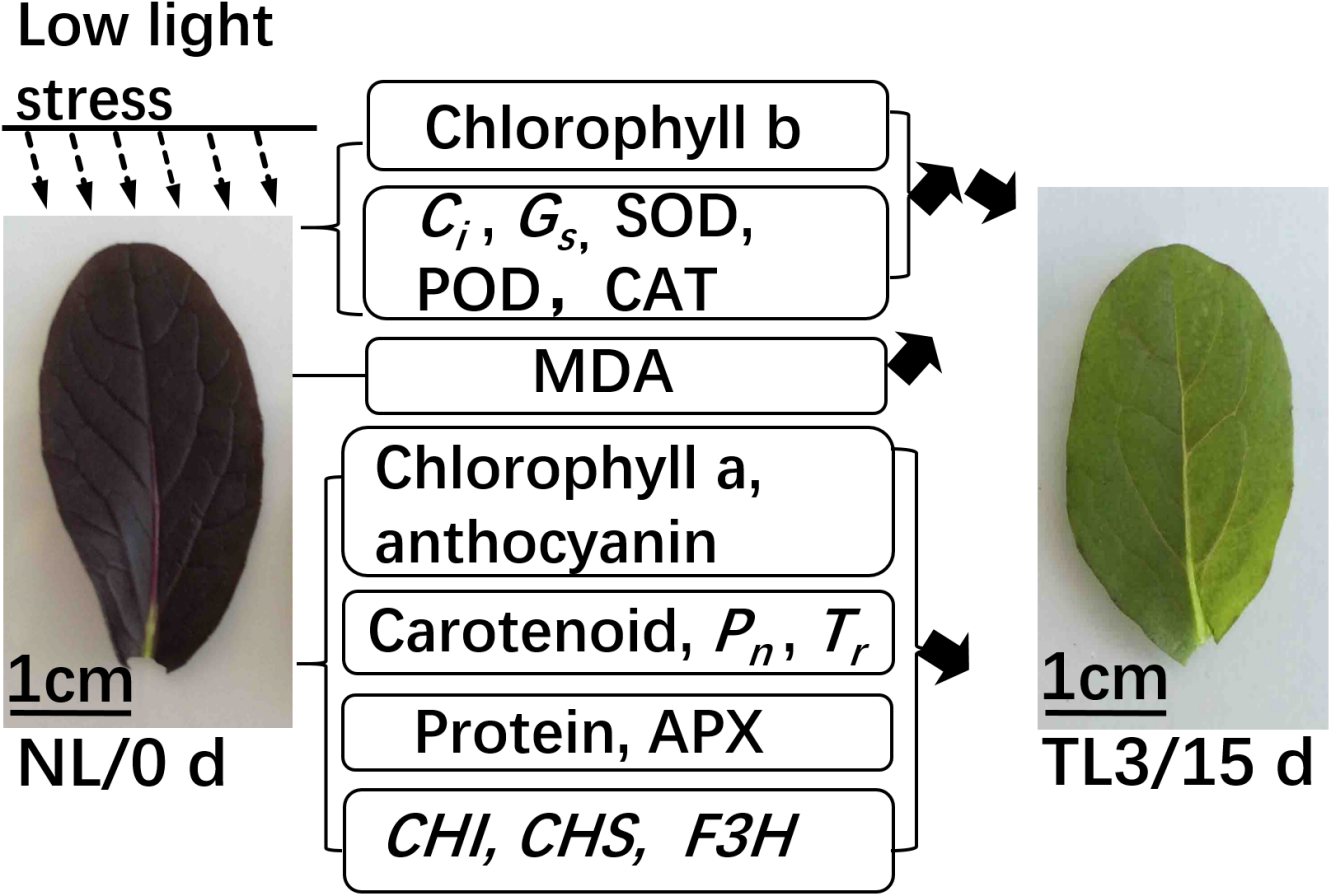
A model of purple pak-choi grown under low light stress. The 45 degree upward sloping black arrow indicates a rise, and the 45 degree downward sloping black arrow indicates a descent.

We summarized our conclusions to a model (Fig 7) to emphasize the physiological changes under light stress. At the early stage of low light treatment, the contents of chlorophyll b, *G*_*s*_, *C*_*i*_, SOD, POD, and CAT were increased to improve photosynthetic efficiency. When the low light stress was extended, it inhibited antioxidant enzyme activity and also suppressed the activity of anthocyanin biosynthesis enzymes, causing purple pak-choi seedlings to fade from purple to green. The physiological mechanism underlying the effects of low light stress on purple pak-choi were elucidated, demonstrating the hazards of low light and providing technical guidance for the cultivation of purple vegetables.

## Supporting information

S1 File The dates used in 5 figures in this paper. (XLSX)
